# A model for the dissemination of circulating tumour cell clusters involving platelet recruitment and a plastic switch between cooperative and individual behaviours

**DOI:** 10.1186/s12862-023-02147-5

**Published:** 2023-08-21

**Authors:** Jorian D. Hapeman, Caroline S. Carneiro, Aurora M. Nedelcu

**Affiliations:** Department of Biology, University of New Brunswick, Fredericton, NB E3B 5A3 Canada

**Keywords:** Cancer, Circulating tumour cell clusters, Metastasis, TGF-β1, Extravasation, Phenotypic plasticity

## Abstract

**Background:**

In spite of extensive research, cancer remains a major health problem worldwide. As cancer progresses, cells acquire traits that allow them to disperse and disseminate to distant locations in the body – a process known as metastasis. While in the vasculature, these cells are referred to as circulating tumour cells (CTCs) and can manifest either as single cells or clusters of cells (i.e., CTC clusters), with the latter being the most aggressive. The increased metastatic potential of CTC clusters is generally associated with cooperative group benefits in terms of survival, including increased resistance to shear stress, anoikis, immune attacks and drugs. However, the adoption of a group phenotype poses a challenge when exiting the vasculature (extravasation) as the large size can hinder the passage through vessel walls. Despite their significant role in the metastatic process, the mechanisms through which CTC clusters extravasate remain largely unknown. Based on the observed in vivo association between CTC clusters and platelets, we hypothesized that cancer cells take advantage of the platelet-derived Transforming Growth Factor Beta 1 (TGF-β1) – a signalling factor that has been widely implicated in many aspects of cancer, to facilitate their own dissemination. To address this possibility, we evaluated the effect of exogenous TGF-β1 on an experimentally evolved non-small cell lung cancer cell line that we previously developed and used to investigate the biology of CTC clusters.

**Results:**

We found that exogenous TGF-β1 induced the dissociation of clusters in suspension into adherent single cells. Once adhered, cells released their own TGF-β1 and were able to individually migrate and invade in the absence of exogenous TGF-β1. Based on these findings we developed a model that involves a TGF-β1-mediated plastic switch between a cooperative phenotype and a single-celled stage that enables the extravasation of CTC clusters.

**Conclusions:**

This model allows for the possibility that therapies can be developed against TGF-β1 signalling components and/or TGF-β1 target genes to suppress the metastatic potential of CTC clusters. Considering the negative impact that metastasis has on cancer prognosis and the lack of therapies against this process, interfering with the ability of CTC clusters to switch between cooperative and individual behaviours could provide new strategies to improve patient survival.

## Introduction

In spite of extensive research efforts, cancer remains a major health problem and the second leading cause of death worldwide [1]. While data on the genetic changes associated with cancer initiation and progression are accumulating, our understanding of how the behaviours of cancer cells are affected by interactions with their microenvironment and surrounding cells is lagging behind. These behaviours are especially relevant to the later stages in cancer progression, including the spread of cells from the primary tumour to other locations in the body – a process known as metastasis. During metastasis, cells first undergo a change in their phenotype from an epithelial state towards a migratory mesenchymal-like state (i.e., the so-called epithelial-mesenchymal transition or EMT [2]). Traditionally, these migratory cells were assumed to individually leave the primary tumour and invade nearby tissue, then enter and travel through the circulatory system (where they are referred to as circulating tumour cells or CTCs), and ultimately disseminate in new tissues and develop into secondary tumours [3]. However, more recently, various types of evidence suggest that tumour cells engage in intra- and/or inter-clonal cooperative interactions during both the migration/invasion steps as well as dispersal through the vasculature [4–9].

The notion of cancer cells cooperating while in the circulatory system is based on the more recent findings of multicellular assemblages of CTCs – referred to as CTC clusters, in the bloodstream of cancer patients with a wide range of malignancies (e.g., cancer of the brain [10], pancreas [11], breast [12], colon [13], kidney [14], liver [15], lung [16] and skin [17]). Furthermore, in spite of their low abundance (2–5% of a typical CTC population [18]), the presence of CTC clusters generally correlates with poor prognosis [19] as their metastatic potential can be up to 50-fold higher than that of single CTCs [16, 18–20]. Many of the factors thought to contribute to the increased metastatic potential of CTC clusters compared to single CTCs are related to general group benefits (i.e., collective/cooperation benefits) in terms of survival [4, 21]. For instance, being in a group is thought to provide cancer cells increased resistance to several challenges in the bloodstream, including shear stress [22], programmed cell death/anoikis [23], immune attacks [24] and drug therapies [25]. Furthermore, it has been shown that CTC clusters feature DNA methylation patterns that promote the expression of stem-cell related genes [26], and that the formation of clusters promotes a metabolic switch that increases the efficiency of metastasis [27].

Despite the obvious advantages of adopting a group behaviour while travelling through the circulatory system, a group phenotype could pose a challenge during the intravasation and extravasation steps (i.e., entering and exiting the vasculature) as the large size would hamper passing through the narrow spaces between the endothelial cells that make up the vessel walls (the so-called transendothelial migration [28]). One way to circumvent this difficulty is for CTC clusters to form via the aggregation of single cells in the vasculature [29]. However, various lines of evidence argue that CTC clusters arise by the cohesive shedding of clusters from the primary tumour (which can be facilitated by hypoxic tumour microenvironments [30]), followed by collective movement into the bloodstream [18, 20]. To account for this possibility, it has been proposed that due to local angiogenesis, the blood vessels that populate a tumour generally have weak cell-cell junctions through which cancer cells, including CTC clusters, could enter the vasculature [28]. Nevertheless, this scenario cannot be applied when cell groups exit the bloodstream to colonize new tissues as the vessels in these tissues are more secure.

The extravasation of single CTCs is thought to require the formation of stable attachments with the endothelial wall [31]. Some studies suggested that single tumour cells will then cross the endothelial barrier in a similar manner to leukocytes, by inserting between endothelial cells and inducing a temporary weakening of the cell-cell junctions (i.e., diapedesis) [32]. Other reports have shown single CTCs to induce either necroptosis in surrounding endothelial cells [33] or vascular remodelling (i.e., angiopellosis) [34], both of which create openings in the vessel wall allowing cells to leave the vasculature. However, it is not clear if/how similar processes can explain the extravasation of CTC clusters. One possibility is that due to their size, CTC clusters become arrested in small capillaries and give rise to metastases through proliferation at their site of arrest [4]. But an in vitro study suggested that CTC clusters can rearrange into single-cell files and could move through thin capillaries [35]. Alternatively, multicellular aggregates caught in capillaries in a zebrafish model have been shown to induce angiopellosis allowing collective extravasation and movement into new tissues [36, 37]. Nevertheless, none of these possibilities have been extensively investigated, and if/how CTC clusters can exit the vasculature and develop into distant metastases, is still not well understood.

CTC clusters typically consist of 2-100 cells organized in grape-like morphologies [38] and often display significant phenotypic heterogeneity, including cells expressing various levels of epithelial and mesenchymal markers [39]. Furthermore, in addition to cancer cells, CTC clusters in the bloodstream are known to contain other cell types such as platelets, endothelial cells, fibroblasts, leukocytes and pericytes [40]. The association of CTC clusters with such cells provides ample opportunity for interactions and crosstalk. Of interest is the close association between platelets and CTC clusters because of the platelets’ ability to release TGF-β1 (Transforming Growth Factor Beta 1) – a signalling molecule that, in addition to its complex roles in normal activities (e.g., platelet-derived TGF-β1 functions in wound healing [41]), also plays a major role in many aspects of human cancers [42]. Notably, it has been shown that single CTCs can activate platelets in mouse models, causing release of TGF-β1, which in turn enhances the metastatic tumour seeding abilities of single CTCs by promoting migratory and invasive properties [43]. Furthermore, a recent study found that 80% of the CTC clusters isolated from patients with pancreatic cancer (especially those with rapid progression in metastasis) were cloaked with platelets [11].

Based on the observed in vivo association between CTC clusters and platelets, we hypothesized that CTC clusters recruit platelets, and the platelet-derived TGF-β1 can facilitate the extravasation and dissemination of CTC clusters into new tissues. To address this possibility, we employed an in vitro model-system that we developed and previously used to investigate the biology of CTC clusters [44, 45]. Specifically, we used an experimentally evolved non-small cell lung cancer line that grows as clusters in suspension (H2122 SS) and evaluated the effect of exogenous TGF-β1 (at a physiologically relevant concentration) on the migration and invasion of cell clusters. Based on our data we developed a new model for the dissemination of CTC clusters that involves a TGF-β1-mediated plastic switch (initiated via the recruitment of platelets) from the cooperative behaviour associated with the dispersal stage to an individual behaviour that allows their extravasation and dissemination.

## Results

### TGF-β1 induces the dissociation of H2122 SS clusters and transition to an adherent mesenchymal-like phenotype

**Fig. 1:**
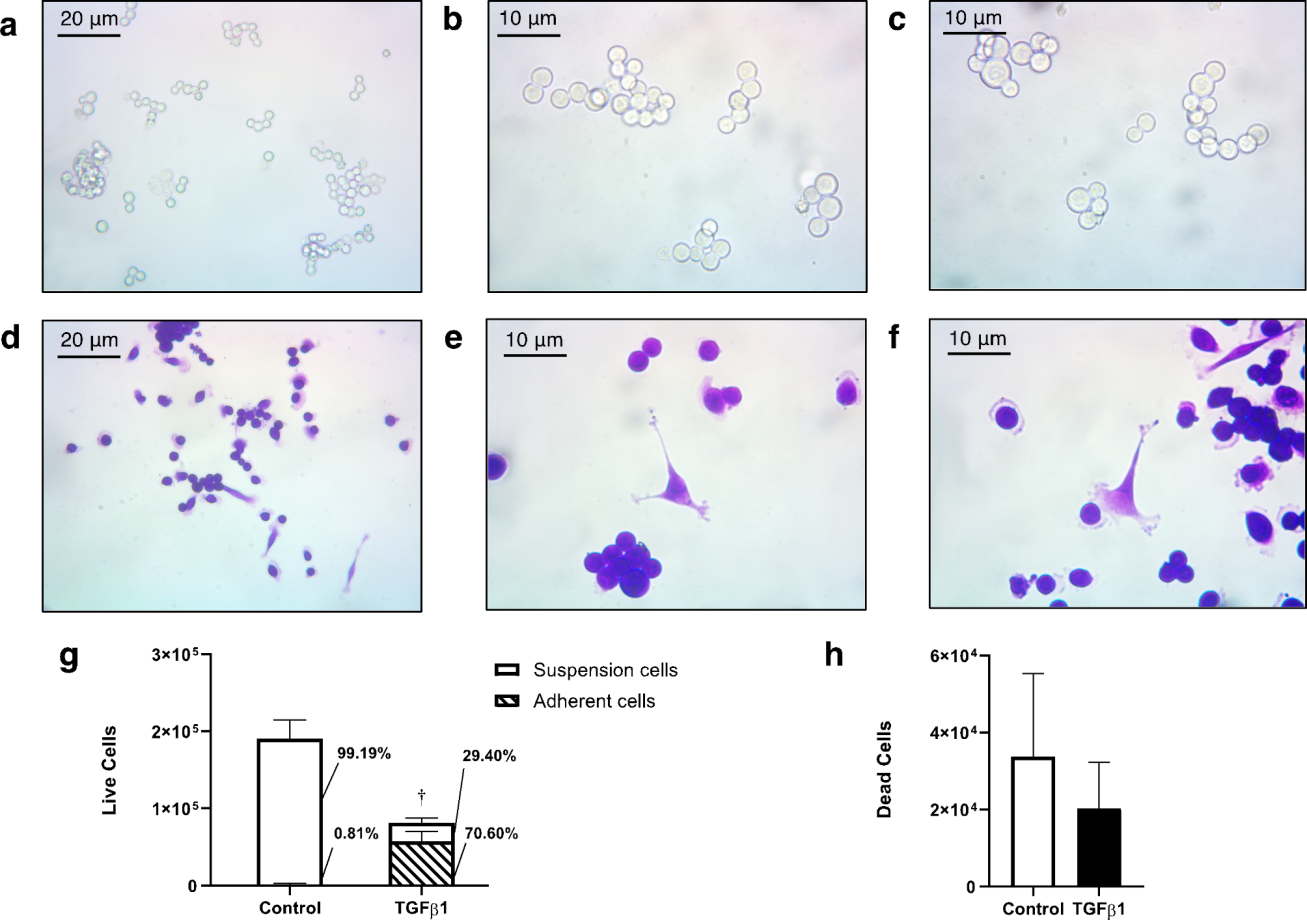
The effect of TGF-β1 on the phenotype of H2122 SS cells. Micrographs comparing the morphology of H2122 SS cells before **(a-c)** and after **(d-f)** exposure to TGF-β1 (cells were stained with 0.2% crystal violet). The number and proportion of adherent and suspension live cells **(g)** and the number of dead cells **(h)** in the H2122 SS cell line treated with 10 ng/ml TGF-β1 and untreated (Control) for 48 h. Error bars represent standard error, n = 3. Cross (†) indicates significant difference in the proportion of adherent cells between the treatment and the control groups (*p* < 0.001; Welch’s two sample t-test)

To address whether TGF-β1 released by recruited platelets could facilitate the extravasation of CTC clusters, we first exposed the H2122 SS cells to TGF-β1 (10 ng/ml) for 48 h, and observed changes in their phenotypic state and morphology. In its native state, the H2122 SS cell line grows as clusters of cells in suspension (Fig. 1a-c). However, following exposure to TGF-β1, most clusters dissociated into single cells that transitioned to an adherent phenotype which often featured cytoplasmic extensions characteristic of mesenchymal cells (Fig. 1d-f).

To quantify this change in phenotypic state, the adherent and suspension cells were counted separately and the proportion of the two cell populations was determined. We found that treatment with TGF-β1 resulted in a significant increase in the proportion of adherent cells (Fig. 1g). Specifically, compared to control cultures, TGF-β1-treated cultures displayed a significantly higher proportion of adherent cells (70.60% vs. 0.81%; *p* < 0.05), indicating that most cells responded to the exogenous TGF-β1 (paracrine signalling) and transitioned to a single-cell adherent state. Interestingly, the treated cultures also showed an overall lower number of live cells (with no change in the number of dead cells – Fig. 1h), indicating that TGF-β1 has an inhibitory effect on cell proliferation.

### TGF-β1 induces the migration of H2122 SS cells

**Fig. 2:**
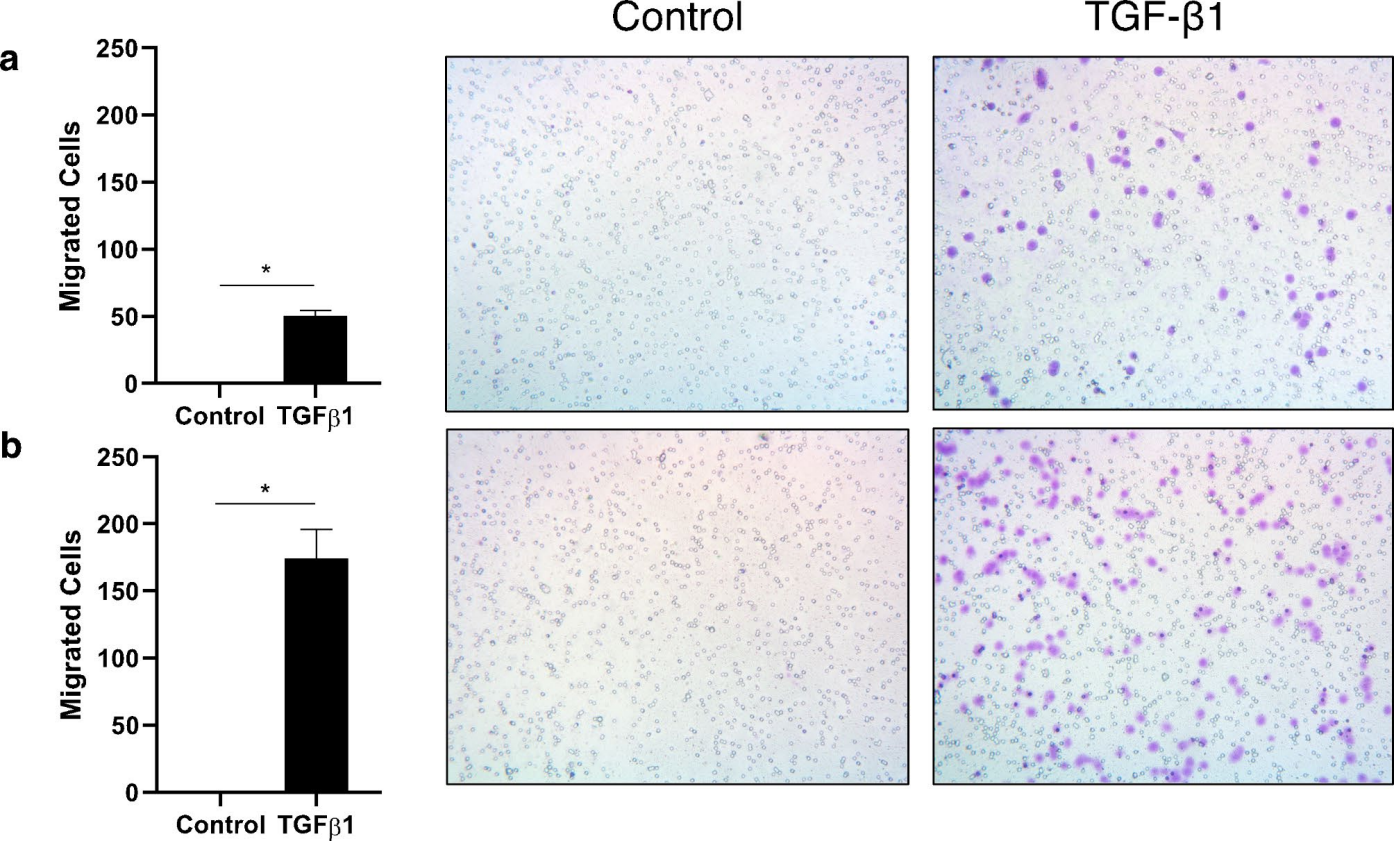
The effect of TGF-β1 on the migration of H2122 SS cells. The number of H2122 SS cells that migrated through a Transwell insert after 24 h **(a)** and 48 h **(b)** post-seeding. Pictures are sample fields of view showing cells (purple dots) that have migrated through the pores (empty circles) and adhered to the underside of the insert (cells were stained with 0.2% crystal violet). Error bars represent standard error, n = 3; asterisks (*) indicate *p* < 0.05 (Welch’s two sample t-test)

To determine whether the morphology of TGF-β1-treated cells reflects an ability to migrate, we used the Transwell assay and assessed the migration of H2122 SS in the presence and absence of TGF-β1, at 24 and 48 h (Fig. 2). Compared to control cultures, significantly more cells migrated in the cultures treated with TGF-β1 (60 cells vs. 0.2 cells per field of view at 24 h, *p* < 0.05; and 174 cells vs. 0.4 cells per field of view at 48 h, *p* < 0.05). Overall, these data indicate that TGF-β1 can also induce the migration of the H2122 SS cells that adhered.

### TGF-β1-treated H2122 SS cultures have increased invasive abilities

**Fig. 3:**
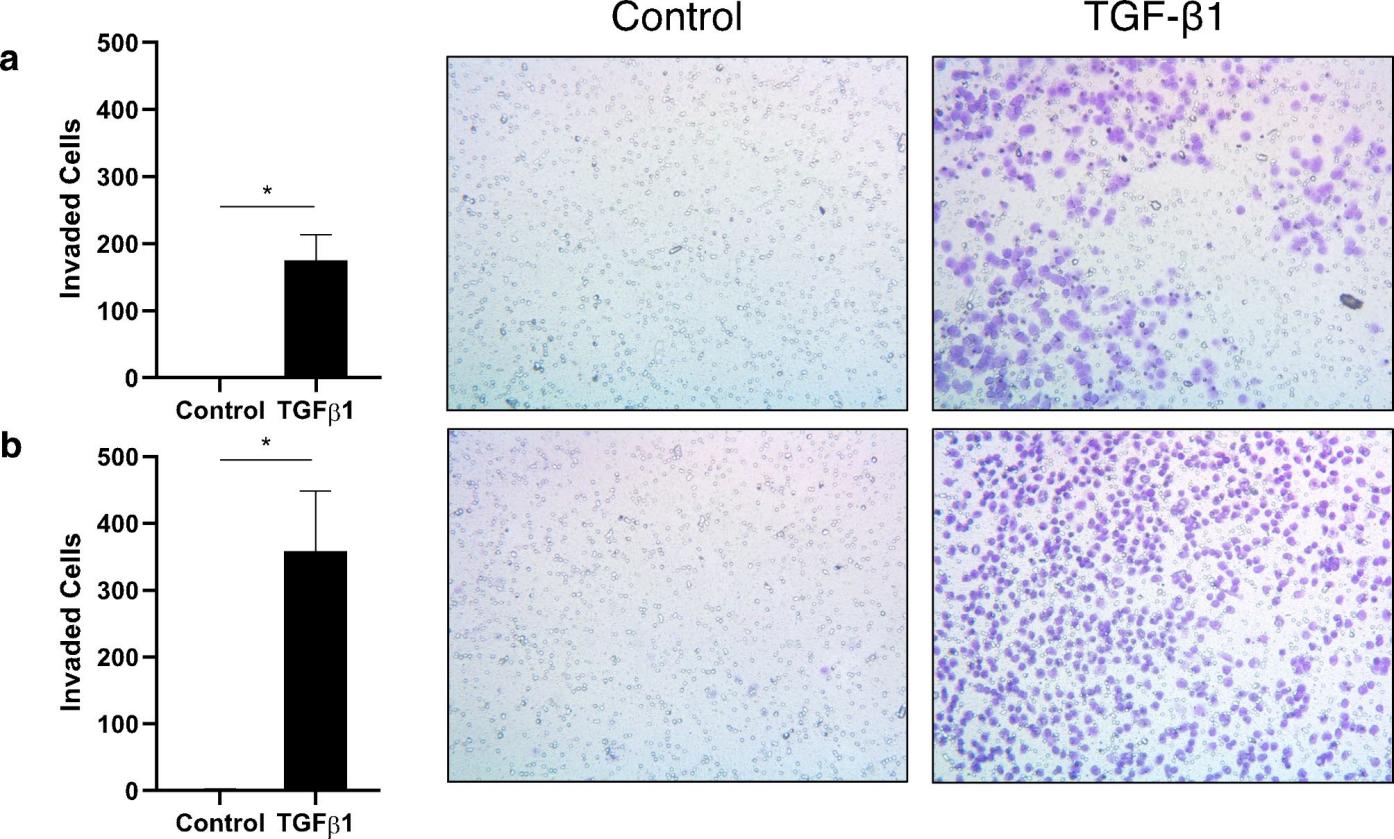
The effect of TGF-β1 on the invasiveness of H2122 SS cells. The number of invaded H2122 SS cells after 48 h **(a)** and 72 h **(b)**. Pictures are sample fields of view showing cells (purple dots) that have migrated through a layer of Matrigel and adhered to the underside of the Transwell insert (cells were stained with 0.2% crystal violet). Error bars represent standard error, n = 3. Asterisks (*) indicate *p* < 0.05 (Welch’s two sample t-test)

We also used the Transwell assay to evaluate the invasive abilities of the H2122 SS line treated with TGF-β1, after 48 and 72 h (Fig. 3). Compared to the control cultures, significantly more cells were able to break down the Matrigel matrix and migrate in the presence of TGF-β1 (176 cells vs. 0.5 cells per field of view at 48 h, *p* < 0.05; 359 cells vs. 1 cell per field of view at 72 h, *p* < 0.05) indicating that TGF-β1 increases the invasive abilities of the H2122 SS line.

### The TGF-β1-induced adherent phenotype is stable in the absence of exogenous TGF-β1

**Fig. 4:**
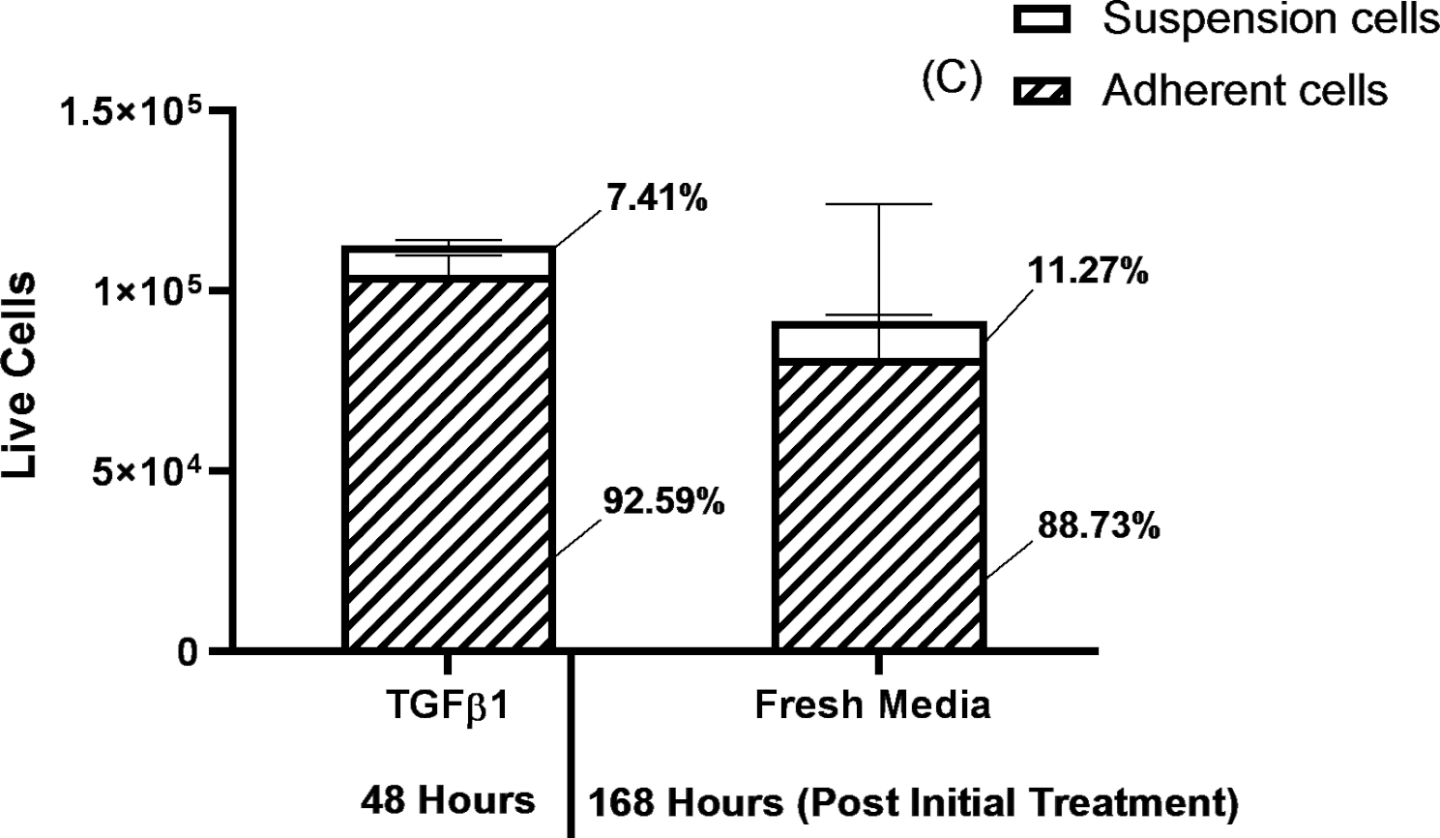
The stability of the TGF-β1-induced adherent phenotype in the absence of exogenous TGF-β1. The number and proportion of live adherent and suspension H2122 SS cells in cultures treated with TGF-β1 for 48 h and cultures grown in the absence of TGF-β1 for an additional 7 days (168 h) after their initial treatment. Error bars represent standard error, n = 3. No significant difference in the proportion of adherent cells in either population (Welch’s two sample t-test)

To evaluate whether the TGF-β1-induced adherent phenotype is stable in the absence of exogenous TGF-β1, we first treated H2122 SS cells with 10 ng/ml TGF-β1 for 48 h to induce the adherent phenotype. We then removed the cells that were still in suspension and assessed the number and proportion of adherent and suspension cells. Next, we washed the adherent cells with PBS and maintained them for another 7 days in RPMI media without TGF-β1 (with media being refreshed every 2 days). After 7 days in the absence of exogenous TGF-β1, we recorded the numbers and proportions of adherent and suspended cells and compared them with those in the population initially treated with TGF-β1 for 48-hours (Fig. 4). Interestingly, compared to the initial cultures treated with TGF-β1, the cultures maintained in media without exogenous TGF-β1 for 7 days did not show a statistically significant difference in the proportion of adherent cells (88.73% vs. 92.59% adherent cells), suggesting that most of the TGF-β1-induced adherent cells do not lose their adherent phenotype in the absence of exogenous TGF-β1. However, although the exogenous TGF-β1 was removed, cells did not resume proliferation.

### TGF-β1-induced adherent cells release TGF-β1

**Fig. 5:**
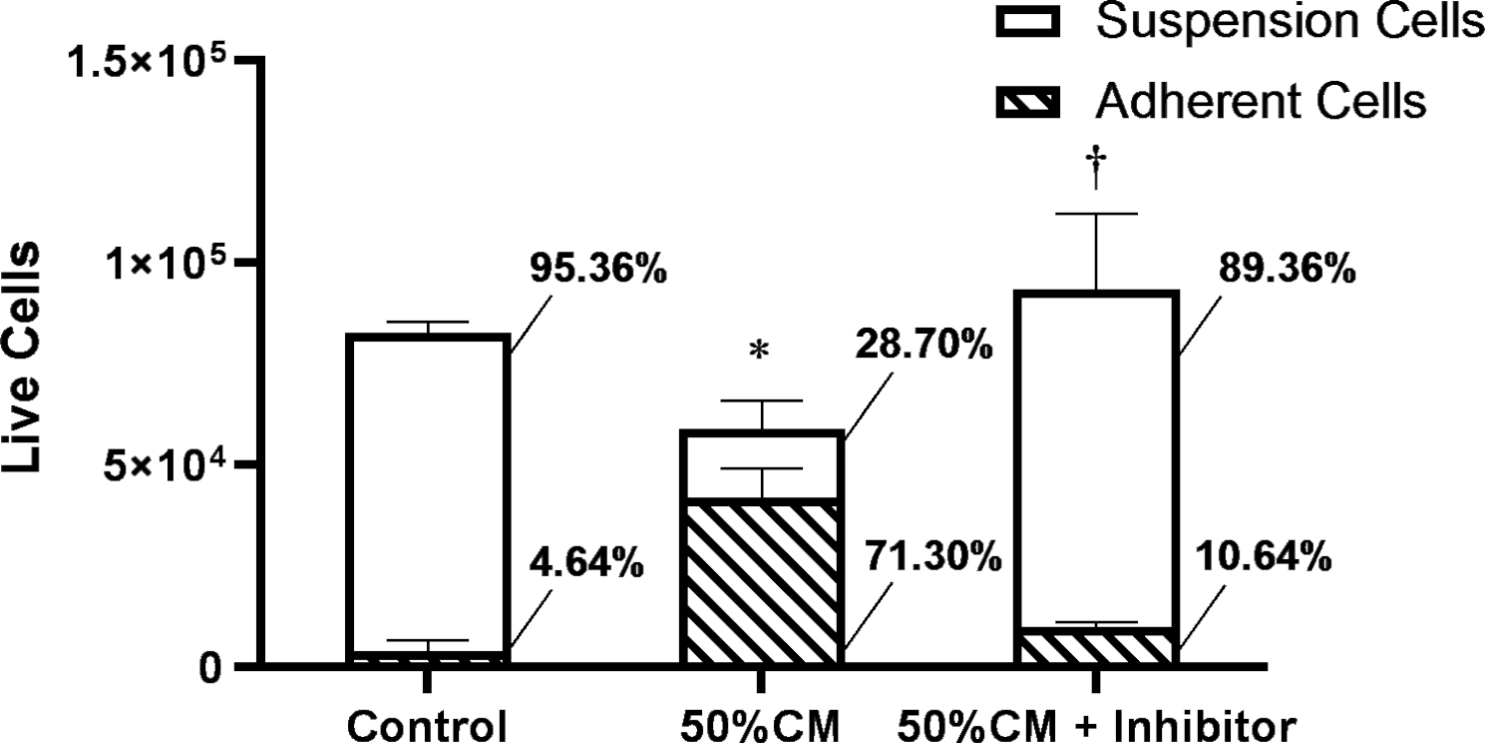
The effect of the conditioned medium from TGF-β1-induced adherent cells on the phenotype of naïve H2122 SS cells. Number and proportion of live adherent and suspension H2122 SS cells in cultures treated with 50% conditioned media (CM) from TGF-β1-induced adherent H2122 SS cells in the absence and presence of 10 µM TGFβRI inhibitor, relative to control. Error bars represent standard error, n = 3. Asterisk (*) denotes a significant difference in the proportion of adherent cells in the total population of the control compared to the 50% conditioned media group and cross (†) indicates significant differences between the 50% CM + inhibitor group compared to the 50% CM group (*p* < 0.001)

To test if the stability of the TGF-β1-induced phenotype and the lack of proliferation might be due to the ability of TGF-β1-induced adherent cells to release and respond to their own TGF-β1 (i.e., autocrine signalling), we collected conditioned media from TGF-β1-induced adherent cells and added it to naïve H2122 SS cells in the presence or absence of a TGFβ1 receptor 1 (TGFβRI) inhibitor. After 48 h, the suspension and adherent cell populations were counted. The addition of conditioned medium (1:1 ratio with fresh medium) resulted in a significant increase in the proportion of adherent cells compared to the control (4.64% in control vs. 71.30% in treatment, *p* < 0.05) (Fig. 5). Furthermore, the addition of the TGFβRI inhibitor (10 µM) significantly decreased the proportion of adherent cells (10.64% in the presence of inhibitor vs. 71.30% without the inhibitor, *p* < 0.05) and restored cell proliferation. Collectively, the data suggest that the ability of the conditioned medium to induce the transition of the naïve SS cells to an adherent phenotype is due to the secretion of TGF-β1 by the adherent cells.

### Autocrine TGF-β1 signalling is not required for the stability of the adherent phenotype

**Fig. 6:**
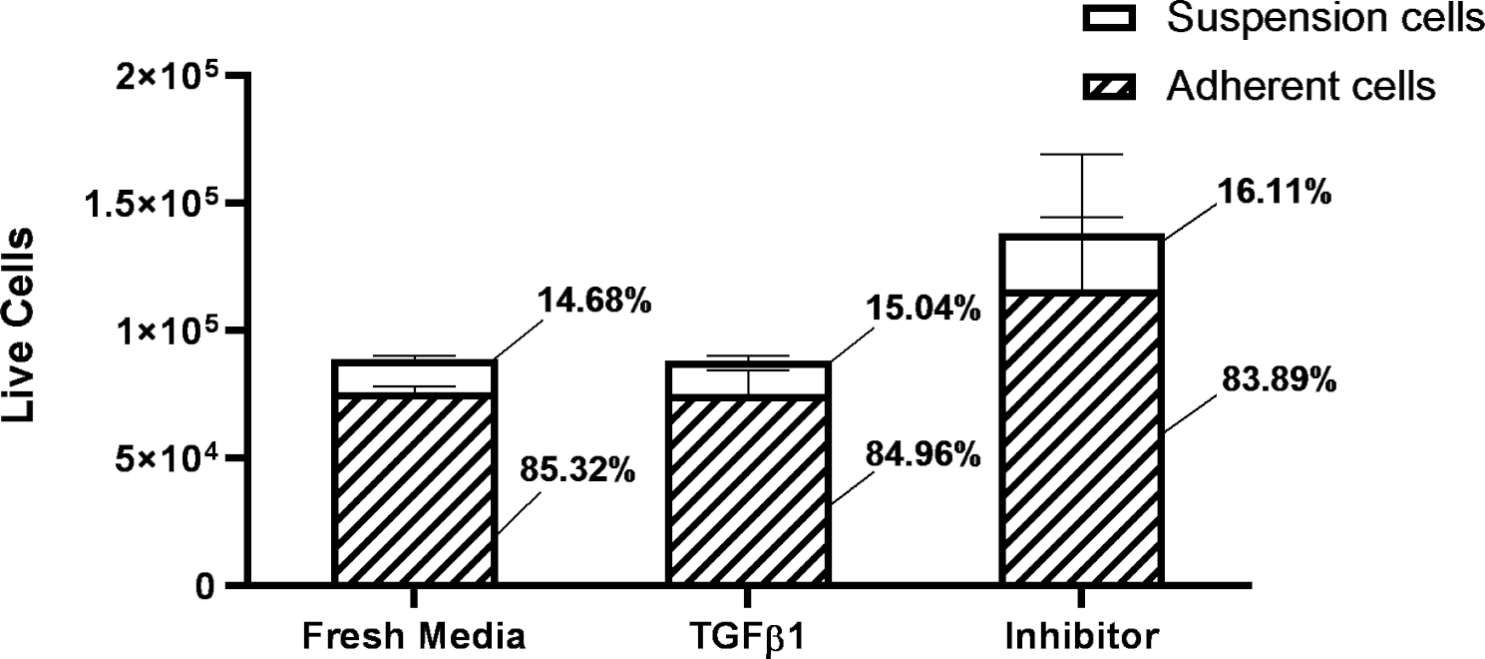
The stability of the TGF-β1-induced adherent phenotype in the absence of autocrine TGF-β1 signalling. Number and proportion of live adherent and suspension H2122 SS cells in TGF-β1-induced adherent cultures following 48 h in fresh media alone or fresh media with either TGF-β1 or a TGFβRI inhibitor. Error bars represent standard error, n = 3. No significant difference in the proportion of adherent cells in either population (One way ANOVA followed by post-hoc Tukey test)

To further address whether the TGF-β1 released by the adherent cells is required to maintain their adherent phenotype, naïve H2122 SS cells were initially treated with 10 ng/ml TGF-β1 for 48 h to induce the adherence. Then, after the adherent cells were washed with PBS, RPMI media alone or media containing either 10 ng/ml TGF-β1 or a TGFβRI inhibitor was added to the adherent cells. After 48 h, the numbers of adherent and suspension cells were assessed and compared. The proportion of adherent cells did not change significantly between the three cultures (Fig. 6). Specifically, in fresh media alone, 85.32% of the cells remained adherent – similar to cultures maintained in medium with exogenous TGF-β1; furthermore, the addition of the TGF-β1 inhibitor did not result in a decrease in the proportion of adherent cells (84.96% and 83.89%, respectively) (Fig. 6). Overall, these data suggest that although, once adhered, H2122 SS cells release their own TGF-β1, autocrine TGF-β1 signalling is not required to maintain the adherent phenotype.

### Autocrine TGF-β1 signalling allows migration in the absence of exogenous TGF-β1

**Fig. 7:**
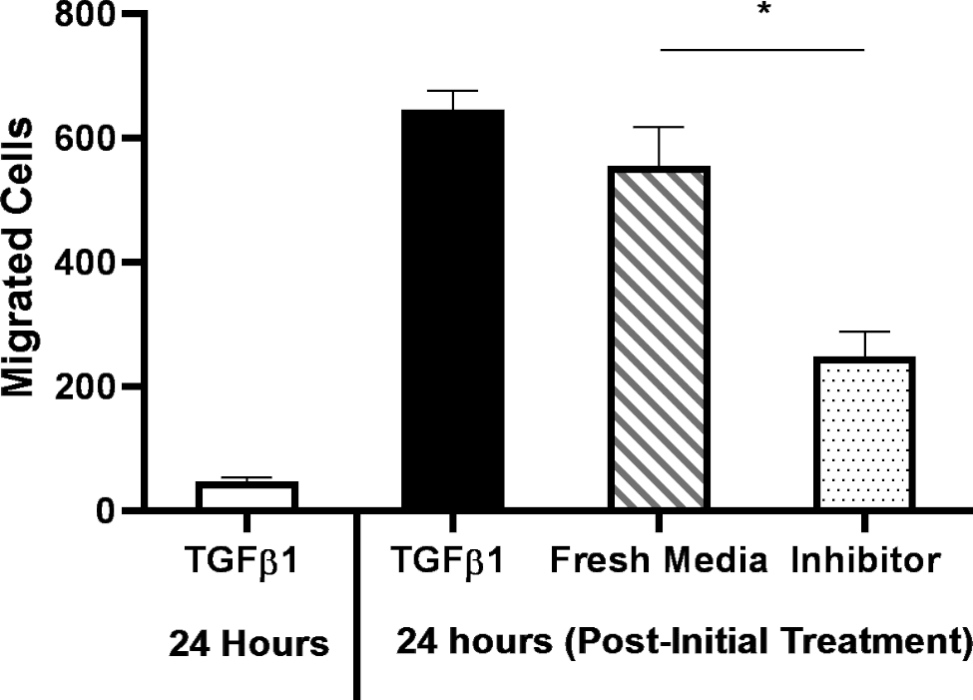
The role of autocrine TGF-β1 signalling in the migration of TGF-β1-induced adherent H2122 cells in the absence of exogenous TGF-β1. Number of TGF-β1-induced adherent H2122 SS cells that migrated to the underside of the insert (per field of view) after the initial treatment with TGF-β1 for 24 h, as well as after an additional 24 h in either the presence of 10 ng/ml TGF-β1, the absence of exogenous TGF-β1, or in the presence of 10 µM TGFβRI inhibitor. Error bars represent standard error, n = 3. Asterisk (*) indicates a significant difference between migrated cells in the absence of exogenous TGF-β1 and the TGFβRI inhibitor (*p* < 0.05)

To investigate the role of the TGF-β1 secreted by the TGF-β1-induced adherent cells, we addressed whether the released TGF-β1 is required for the migration of the adherent cells in the absence of exogenous TGF-β1. H2122 SS cells were seeded in Transwell inserts in the presence of TGF-β1 to induce the adherent phenotype within the insert, and cell migration was evaluated after 24 h. Then, after the adherent cells on the top side of the inserts were washed with PBS, either RPMI media, RPMI media with TGF-β1, or RPMI media with a TGFβRI inhibitor was added to the inserts. The inserts were subsequently placed in a new well, and the adherent cells were allowed to migrate for another 24 h, before the migration abilities of each group were assessed (Fig. 7). Cells in RPMI media without exogenous TGF-β1 migrated as much as cells in the presence of TGF-β1, suggesting that they were able to secrete and respond to their own TGF-β1 (autocrine signalling). Furthermore, the addition of a TGFβRI inhibitor reduced the number of cells that migrated, confirming that autocrine TGF-β1 signalling plays a role in maintaining a migratory state in the absence of exogenous TGF-β1.

## Discussion

### A model for the dissemination of CTC clusters involving platelet recruitment and TGF-β1 signalling

The goal of this study was to address the hypothesis that CTC clusters can recruit platelets to facilitate their extravasation through platelet-derived TGF-β1. This possibility is consistent with the fact that RNA sequencing revealed strong levels of TGF-β1 signatures in human breast CTC clusters coated with platelets [46]. To mimic CTC clusters, we used an experimentally evolved lung cancer cell line that grows as non-adherent clusters with similarities to real CTC clusters in terms of size, cell-cell connections, and expression of mesenchymal and epithelial markers [44].

To simulate the presence of platelets, we used exogenous TGF-β1 at a concentration of 10 ng/ml (but concentrations as low as 1 ng/ml were also effective; our unpublished data). Reported TGF-β1 levels in human plasma vary greatly (from 0.1 ng/ml to more than 25 ng/ml), with ca. 4 ± 2 ng/ml being the most common [47, 48]. However, the presence of platelets can greatly increase these levels as platelets contain between 2,500 and 4,000 TGF-β1 molecules/cell and contribute up to 45 ng TGF-β1 per 1 ml of blood [47, 49]. Furthermore, TGF-β1 levels might increase during cancer progression, as elevated TGF-β1 levels were reported in patients with advanced breast cancer [50].

**Fig. 8:**
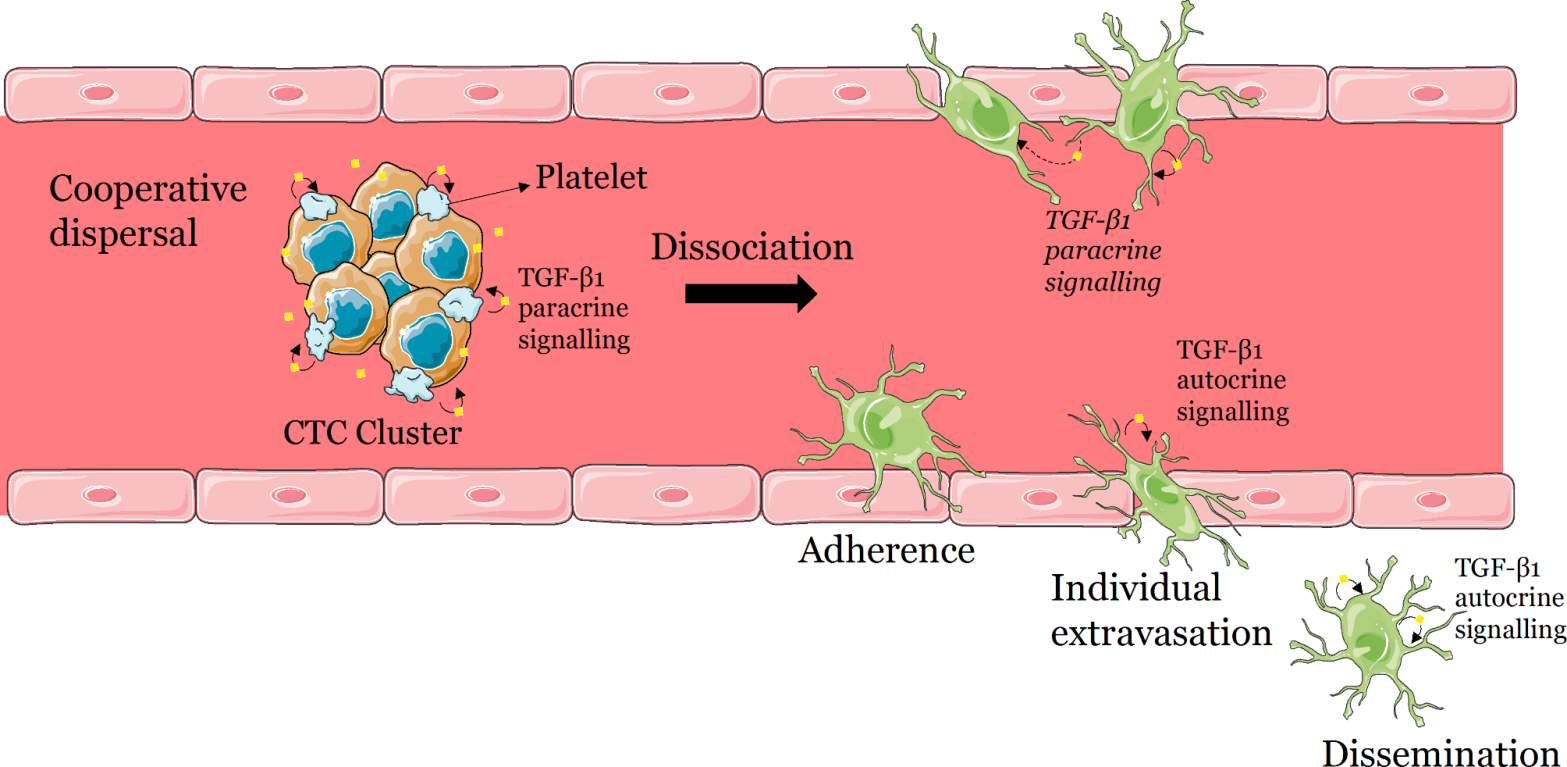
Proposed mechanism for the extravasation and dissemination of CTC clusters involving a switch from a cooperative phenotype to a single-celled stage (mediated by platelet-derived TGF-β1; paracrine signalling) capable of extravasation and dissemination (involving autocrine TGF-β1 signalling). In the bloodstream, CTC clusters recruit platelets that become attached to the cluster. In response to the TGF-β1 released from platelets (paracrine signalling), CTC clusters dissociate and individual CTCs adhere to the endothelial wall. Once adherent, these cells secrete their own TGF-β1 that induces a mesenchymal-like state (autocrine signalling) with invasion capabilities, which allows them (at least initially) to individually extravasate and disseminate into the adjacent tissues. Paracrine signalling among nearby individual CTCs is also possible, if clusters are composed of cells that differ in their ability to secrete TGF-β1. Lastly, although not shown here, extravasated cells could also aggregate through homotypic interactions in the perivascular space and/or undergo collective migration and invasion (see text for discussion). Diagram created with Servier Medical Art at www.smart.servier.com

Using our in vitro model-system, we first observed that TGF-β1 induced a switch from a suspension cell-cluster phenotype to an adherent mostly single-cell state. Importantly, once adhered, these cells do not require TGF-β1 (from either an exogeneous source or self-produced) to maintain their adherent state. In addition, while cell clusters have almost no migration and invasion potential in the absence of TGF-β1, the TGF-β1-treated cells have greatly enhanced abilities to migrate and invade relative to the naïve cell clusters. Furthermore, once adherent, the cells produce and release their own TGF-β1 that can induce a migratory behaviour in the absence of exogenous TGF-β1. Based on these findings, below we propose and discuss a step-by-step model for the extravasation and dissemination of CTC clusters in which platelet-derived TGF-β1 induces the dissociation of clusters into individual adherent cells that secrete their own TGF-β1, which allows them to extravasate and disseminate into nearby tissues (Fig. 8).

### Platelet TGF-β1-induced CTC cluster dissociation and adherence

Based on our finding that upon treatment with TGF-β1, the H2122 SS cell clusters dissociate and develop an adherent phenotype, we suggest that in the bloodstream, crosstalk between platelets and CTC clusters may cause a TGF-β1-mediated transition from a cooperative phenotype to an individual adherent stage (Fig. 8). Since TGF-β1 is known to be involved in inducing EMT by downregulating the expression of cell-cell adhesion proteins such as E-cadherin [51], it is possible that the downregulation of such proteins may also be responsible for the dissociation of H2122 SS cell clusters into single cells after treatment with TGF-β1.

Adhesion to the endothelial wall is an important step in CTCs dissemination; in fact, it was suggested that such cell-cell interactions, and not mechanical entrapment, are responsible for the arrest of CTCs in microvessels [52]. Nevertheless, a role for TGF-β1 in inducing adherence to a substrate is relatively less known. However, in certain cell lines, TGF-β1 has been described as being involved in the expression of proteins that promote adhesion to the extracellular matrix during migration [53, 54]. Furthermore, a similar response to what we observed has been reported in a gastric cancer cell line that grows predominantly as clusters in suspension; in this case, the TGF-β1-induced adherence (at as low as 1 ng/ml) was found to involve the expression of integrin alpha-3 subunit [55]. Given that H2122 SS is derived from a cell line that grows as a mixture of adherent and suspension cells, it is possible that this evolved line still maintains the ability to express an adherent phenotype under specific conditions. Overall, this predisposition towards an adherent phenotype is not unlike CTC clusters in vivo as CTC clusters isolated from the bloodstream and cultured in treated tissue flasks often transition to an adherent phenotype [56].

Although the attached cells might initially remain in contact with platelets and the associated TGF-β1, we found that the adherent phenotype can be stable in the absence of any source (exogenous or self-produced) of TGF-β1. One possibility is that the stability of the phenotype is the result of trans-generational epigenetic changes. In fact, it has been suggested that TGF-β1-induced EMT can involve epigenetic changes. For example, TGF-β1 treatment of certain lung cancer cell lines results in EMT being induced in part by demethylation of *Slug*, a gene coding for a prominent EMT-inducing transcription factor [51]. Alternatively, there is evidence that cell-substratum adhesion in some cell lines is mediated by intracellular feedback regulation stimulated by integrin-substrate interaction [57]. Therefore, the adherent phenotype’s stability even in the absence of TGF-β1 may be the result of positive feedback loops responding to the initial TGF-β1-induced attachment to the substrate. Another possibility involves intracrine TGF-β1 signalling, whereby latent TGF-β1 is activated intracellularly [58] and through a “private” loop [59] active TGF-β1 maintain its own expression and the expression of the pathways it regulates.

### TGF-β1-induced single-celled extravasation

Based on the TGF-β1-induced migratory and invasive abilities seen in vitro, we suggest that, in response to TGF-β1, the cells that adhered to the endothelial wall can migrate/invade as single cells through the endothelium into new tissues (Fig. 8). TGF-β1 is known to induce EMT and has been shown to increase migration and invasion in several adherent cancer cell lines such as breast [60], lung [51] and colon [61], among others. Interestingly, a link between TGF-β1 and the switch from collective to single cell motility (albeit in adherent state) has already been reported. Specifically, using a breast cancer cell line that grows as distinct adherent colonies when seeded at low density, Giampieri et al. showed that treatment with TGF-β1 caused the cells to dissociate and migrate as single cells both in vitro and in vivo [62].

The dissociation of clusters into single cells would allow for easier passage through the endothelial wall and prevent the need for very large openings in the blood vessel. In the single cell state, cells could extravasate using any of the known mechanisms proposed for single CTCs, such as through diapedesis [32] by inducing necroptosis [33], or by causing vascular remodelling through angiopellosis [34]. Overall, significant remodelling of the vasculature would not be necessary because there is no need for the entire cluster to move through the opening at once. If CTC clusters dissociate at the site of extravasation, tumour cells could still benefit from travelling through the vasculature as a group, but also exploit the benefits of extravasating as single cells through the endothelial wall. However, our model does not exclude the possibility that following extravasation, cells can aggregate through homotypic interactions in the perivascular space, in the same way as certain aggregative CTC clusters have been found to form prior to intravasation in some models [29] and/or engage in collective and cooperative migration and invasion behaviours similar to those proposed to take place during detachment from the primary tumours (e.g., [63, 64]).

### Autocrine TGF-β1 signalling-mediated extravasation and dissemination

The initial steps in the migration and invasion of single cells during extravasation might be mediated by platelet-derived TGF-β1 if the association with platelets is maintained after adherence. However, once cells exit the bloodstream, the limited availability of TGF-β1 in tissues (e.g., [65]) can restrict their migration/invasion capabilities (although some tissues – especially bones, can contain high levels of TGF-β1; [66]). Based on the fact that TGF-β1-induced adherent cells secrete their own TGF-β1 (which allows them to migrate in the absence of exogenous TGF-β1), we suggest that a similar TGF-β1 autocrine signalling ensures that once CTCs leave the bloodstream (and separate from the platelet-derived TGF-β1) they can maintain (at least initially) their migratory/invasive capabilities on their own until reaching appropriate sites for proliferation. Remarkably, TGF-β1 autocrine loops have been shown to last for up to 10 population doublings in the absence of exogenous signals (e.g., [67]).

TGF-β1 autocrine signalling is known to be important in cancer. For instance, in certain types of breast cancer, invasiveness and tumour progression are promoted by autocrine TGF-β1 signalling [68, 69]. Similarly, the mesenchymal state of MCF10A (a non-tumorigenic human epithelial cell line) is maintained through an autocrine mechanism involving TGF-β1, Snail1 and miR-200 [70]. As TGF-β1 is known to induce its own expression [71, 72], we suggest that in our model, the autocrine loop is initiated by the platelet-derived TGF-β1 (Fig. 8).

Interestingly, we also found that adherent cells in the presence of either exogenous or self-produced TGF-β1 have a low proliferation potential. This is consistent with known anti-proliferative effects of TGF-β1 [73] and the proliferation-migration trade-off thought to characterize cancer cells [74]. Obviously, to develop into secondary tumours, cancer cells need to proliferate, which will likely require to discontinue the release of, and/or response to, TGF-β1 (and a switch to an epithelial state). This could be achieved in response to tissue microenvironmental cues since TGF-β1 signalling can be regulated by various feedback loops that provide versatile and context-dependent functions [75].

### CTC dispersal and dissemination involves both cooperative and individual behaviours

Although cancer progression is predicated on selection acting at the cell level, the possibility that cancer cells can engage in cooperative interactions/behaviours that can increase their fitness should also be addressed [76]. Such phenomena have been suggested to facilitate the early metastatic process from inducing EMT in non-metastatic cells to collective migration (with leading and follower cells) and cooperative invasion [5, 77, 78]. The association of CTCs in multicellular aggregates and the advantages associated with this phenotype suggest that cancer cells can also engage in cooperative behaviours during the dispersal stage [4, 6, 21–25].

However, as with other multicellular phenotypes, benefits can be counteracted by costs in some specific contexts or conditions. For instance, a large size can be beneficial in terms of avoiding predation but can be costly in terms of growth or reproduction. In order to fully realize the benefits of cooperation, various additional strategies have to evolve, some of which involve transitory phenotypic changes as part of the life cycle. In the case of CTC clusters, the dissociation of multicellular aggregates into single cells with migratory capabilities can be understood as such a change that allows cancer cells to take advantage of the benefits of being in a group during the passive dispersal through the vasculature but still be able to actively extravasate as single cells.

In this framework, the overall dispersal/dissemination strategy adopted by cancer cells is rather similar to the life cycle of dictyostelid social amoebae [79]. Specifically, under food limitation, single-celled soil amoebae switch to a cooperative behaviour resulting in the formation of a multicellular motile slug (with leading and trailing cells) that migrates to the soil surface. The slug – which includes cells in different physiological states (but also different genotypes), then reorganizes and develops into a fruiting body composed of non-reproductive altruistic cells (differentiated from the leading cells) and single-celled reproductive spores (derived from the followers). The single-cell spores disseminate and are capable of proliferation once they reach a favourable site. This life cycle expresses both cooperative behaviours (i.e., collective migration as a slug and division of labour during multicellular development and spore formation) and individual behaviours (during the single-celled amoeba and spore stages) whose expression involves extracellular signalling molecules and is dependent on environmental cues.

Similarly, in advanced primary tumours distinct clones and/or phenotypes (i.e., cells expressing either epithelial or mesenchymal markers) with different migration/invasion abilities can engage in cooperative behaviours during the early metastatic stages (collective migration and invasion) [77, 80] resulting in heterogeneous CTC clusters. For instance, mesenchymal-like cells (i.e., cells that have undergone EMT) are thought to facilitate the access of non-EMT cells into bloodstream by either acting as leaders or inducing EMT in non-EMT cells [64, 77, 81, 82]. In our model, the dissemination step requires the passive transport of clusters through vasculature, followed by the dissociation of clusters and the extravasation as single cells. Nevertheless, cooperative interactions might still occur during this step. For instance, clusters containing both proliferative and invasive cells have been isolated from melanoma patients, and – at least in a zebrafish model, cooperation between the two cell types has been shown to facilitate the dissemination of proliferative cells and seeding of metastasis [7]. Specifically, during extravasation, clusters reorganize and proliferative and invasive cells co-extravasate, with the invasive cells leading the way [7].

Given the heterogenous nature of the clusters in the H2122 SS line (with respect to the expression of mesenchymal and epithelial markers; [44]) it will be of interest to address whether cooperative interactions might also take place during extravasation and seeding in our model-system. For instance, it is possible that not all cells can release TGF-β1 once attached; but those that cannot might be able to respond (paracrine signalling) and express a migratory and invasive behaviour if they are in the vicinity of TGF-β1 producers. Such cooperative interactions are generally envisioned in primary metastatic tumours during local migration/invasion, but they could be re-expressed during the dissemination step and account for the observed polyclonal composition of secondary tumours (thought to reflect the polyclonal nature of both CTC clusters and primary tumours) [20].

### Clinical implications and limitations

Despite increasing research efforts, the five-year survival rate for most patients with metastatic cancer remains distressingly low [83]. Our inability to improve survival rates underscores the need for a deeper understanding of the metastatic process as well as for developing strategies to suppress it. The significance of CTC clusters in metastatic disease is becoming increasingly recognized and elucidating the mechanisms involved in their extravasation and dissemination has the potential to yield new therapeutic targets.

Our model allows for the possibility that new therapeutic strategies can be developed against TGF-β1 signalling components and/or TGF-β1 target genes to slow the development of metastatic tumours by specifically inhibiting steps in the extravasation and dissemination of CTC clusters. Of interest are recent reports that metformin (a drug used to treat type 2 diabetes but that is intensively studied for its potential anti-cancer effects) can interfere with both the secretion of and the response to TGF-β1 as well as the TGF-β1 itself [84–86]. Additionally, if the expression of TGF-β1 receptors correlates with the metastatic potential of CTC clusters, specific markers with prognostic and therapeutic value could be developed. However, studies in other in vitro systems are needed to confirm this model. Specifically, we expect similar results in cell lines that express the TFG-β1 receptor and do not have mutations in components of the TGF-β1 signalling pathways. Furthermore, in vivo studies are required to evaluate whether TGF-β1 signalling components can be used as potential therapeutic targets to suppress the metastatic abilities of certain types of CTC clusters.

## Conclusion

Our study provides in vitro experimental evidence for a new model for the extravasation and dissemination of CTC clusters involving cluster dissociation and adherence mediated by crosstalk between platelets and cancer cells, followed by single-cell extravasation and migration facilitated by autocrine TGF-β1 signalling (Fig. 8). Current models propose that CTC clusters are arrested in small capillaries and either proliferate at their site of arrest [4] or remodel the surrounding vasculature and exit as multicellular clusters [36]. These are all viable possibilities, and we suggest that given the complexity and diversity of cancer, multiple mechanisms might be at play. That is, CTC clusters may make use of any one of these extravasation and dissemination mechanisms depending on cancer type, the composition of CTC clusters in terms of cancer cell phenotypes (i.e., epithelial, mesenchymal, or mixed) and non-cancer cells types (e.g., platelets), the expression of certain receptors (e.g., TGF-β1 receptors), their location (blood vs. lymphatic vessels) etc. Our proposed model applies specifically to CTC clusters that enter the vasculature as cohesive units, travel through bloodstream, interact with platelets and can respond to TGF-β1 (i.e., express TGF-β1 receptors) in both a paracrine and autocrine context. Considering the lack of therapies that directly affect metastasis, interfering with the plastic phenotypic switches associated with changes in cancer cell behaviour as part of the general dispersal/dissemination strategy exhibited by CTC clusters could provide new ways to improve survival rates.

## Materials and methods

### Cell line and culture conditions

We used an experimentally evolved cancer cell line derived from a non-small cell lung cancer cell line obtained from the American Type Culture Collection – ATCC (Manassas, VA, USA). This original cell line (NCI-H2122) was established from the pleural effusion of a 46-year-old female with stage-4 adenocarcinoma and grows as a mixture of two distinct phenotypes: clusters of cells growing in suspension and adherent cells that grow as a monolayer. By selectively passaging only the adherent or suspension cell populations, two cell lines were experimentally evolved: one that grows as cell clusters in suspension (referred to as the H2122 Suspension-Selected or SS line) and one that grows as adherent cells [45]. The SS line has been previously shown to be a viable model-system for CTC clusters [44]. The SS cells were grown at 37 °C and 5% CO_2_ in RPMI-1640 media (MP Biomedicals) supplemented with 10% FBS and 1% Penicillin/Streptomycin mix. The concentrations of glucose and glutamine in the media were adjusted to 5 mM and 0.5 mM respectively, to simulate physiological levels. Cells were passaged every 2–3 days at a 1:3, 1:4 or 1:5 ratio depending on the required cell density.

### General experimental set-up

Cultures were seeded (in triplicates) in tissue-treated 12-well plates (Sarstedt) at a density of 1 × 10^5^ cells/ml for all experiments (except for Transwell assays – see below, and the conditioned media experiments in which cells were seeded at 5 × 10^4^ cells/ml – see results). Several independent trials were conducted for each experiment.

### Reagents

Human recombinant TGF-β1 (R&D Systems − 7754-BH-005) was reconstituted in 4 mM HCl at 10 mg/ml and was added directly to the culture medium at 10 ng/ml. A TGF-β receptor 1 (TGFβRI) inhibitor (EMD Millipore − 616464) was used at a concentration of 10 µM.

### Cell counting and viability assessment

Cells were stained with Syto-9 (3.34 mM in DMSO, Invitrogen) and Propidium Iodide (20 mM in DMSO, Invitrogen) at a final concentration of 10 µM and 60 µM, respectively. Numbers of live and dead cells were assessed (4 technical replicates for each biological replicate) using the Countess™ II FL Automated Cell Counter (Invitrogen). The suspension and adherent cell populations for each biological replicate were counted separately.

#### Conditioned media collection

To collect conditioned media from TGF-β1-treated cells, naïve SS cells were placed in a 12-well plate at 1 × 10^5^ cells/ml and treated with TGF-β1 for 48 h. The media was removed, the adherent cells were washed with PBS, and fresh media containing 1% FBS was added to the cells. After 24 h, this conditioned medium was collected, centrifuged at 1000xg for 10 min, and the supernatant was filter sterilized before being used in experiments at 1:1 ratio with fresh medium.

### Transwell assays

Cell migration and invasion were assessed using the Transwell assay [87, 88]. Cells were suspended in FBS-deficient RPMI media at 1 × 10^6^ cells/ml, and aliquots of 100 µl were added to 6.5 mm Transwell inserts (Corning). For invasion, 100 µl of Matrigel (Corning − 354277) (1.5 mg/ml) was first added to the inserts and allowed to solidify for 8 h.

The inserts were each placed into individual wells of 24-well plates containing RPMI-1640 media with FBS, which was used as chemoattractant. Cells were then allowed to migrate/invade through the membrane towards the chemoattractant media for 48 or 72 h and the cells trapped on the membrane were fixed and stained (see below). Stained membranes were then visualized at 100x magnification, and 10 random fields of view were photographed. The number of cells for each photo was assessed using the ImageJ cell counter tool [89] and the average number of cells migrated was calculated.

#### Staining adherent cells and transwell inserts

TGF-β1-induced adherent cells and cells attached to Transwell inserts were initially fixed to their substrate by coating them with 70% ethanol for 5 min after which the ethanol was removed, and the cells were dried for 15 min. They were then stained with a 0.2% crystal violet solution (in 20% ethanol) for 10 min, washed with PBS five times and imaged [90, 91].

#### Microscopy and image processing

Photos were taken with an OMAX USB digital microscope camera. Background noise was removed using the Calculator plus tool in FIJI [89] and the smear tool available in the GIMP 2.10 software.

#### Statistical analyses

Statistical analyses were done using GraphPad Prism 8.0.2. The average of each group was calculated from three replicates and expressed as mean ± SE. Welch’s two sample t-test was used to assess statistical significance for differences between two groups, while ANOVA was used for 3 or more groups. A difference between groups was considered statistically significant at *p* < 0.05.

## Data Availability

The datasets generated and/or analysed during the current study are available in the Zenodo repository, https://zenodo.org/record/7135132.
